# Changes in Fatty Acid and Volatile Compound Profiles during Storage of Smoked Cheese Made from the Milk of Native Polish Cow Breeds Raised in the Low Beskids

**DOI:** 10.3390/ani10112103

**Published:** 2020-11-12

**Authors:** Patrycja Dopieralska, Joanna Barłowska, Anna Teter, Jolanta Król, Aneta Brodziak, Piotr Domaradzki

**Affiliations:** Institute of Quality Assessment and Processing of Animal Products, Faculty of Animal Sciences and Bioeconomy, University of Life Sciences in Lublin, Akademicka 13, 20-950 Lublin, Poland; patrycja.dopieralska@interia.eu (P.D.); jolanta.krol@up.lublin.pl (J.K.); aneta.brodziak@up.lublin.pl (A.B.); piotr.domaradzki@up.lublin.pl (P.D.)

**Keywords:** cheese, smoking, storage, fatty acids, volatile compounds, vacuum-packing, native cattle breeds

## Abstract

**Simple Summary:**

Cheese, due to its high nutritional value, is an important element of the daily diet of many consumers. About one third of cow milk produced globally is processed into a wide assortment of cheeses. Most of them are produced on a mass scale in industrial conditions. One factor determining cheese quality is the quality of the milk it is made from. The milk of pasture-grazed cows is known to have higher content of compounds benefiting human health. Consumers are increasingly interested in artisanal products, including cheese, manufactured directly on farms or in small, local processing facilities, regarding them as natural, less processed, and free of additives. Milk for the production of this type of cheese usually comes from cows of native breeds kept on family farms. Smoking is one of the oldest traditional methods used to prolong the shelf-life of food. It imparts a pleasant aroma to cheese and improves its palatability. The literature lacks studies on the quality of smoked cheese during storage. The aim of this study is to assess changes in chemical composition and in fatty acid and volatile compound profiles during storage of smoked cheese made from the milk of native Polish cow breeds.

**Abstract:**

This study investigated changes in the proximate chemical composition and profiles of fatty acids and volatile compounds of 12 smoked cheeses made from the milk of native Polish cow breeds used in Beskid Niski. Analyses were performed during the shelf life i.e., in the 1st, 21st, 42nd and 69th day of storage. Studies have shown that thanks to smoking and vacuum-packing, the chemical composition of cheese remained stable throughout the whole shelf-life. Up until the 21st day of storage, there were no statistically significant changes in the profile of fatty acids as well as volatile compounds. Changes were observed only after the mentioned storage time. After 21 days, there was a significant (*p* < 0.05) and steady decrease (up to day 69) in the proportion of odd-chain (by about 36%), branched-chain (by about 17%) and unsaturated fatty acids (by slightly over 1%). Among unsaturated fatty acids (*p* < 0.05), however, there was a significant increase in the proportion of monounsaturated fatty acids (by 5%) and a decrease in polyunsaturated fatty acids of nearly 12%. Storage lowered (by 47% in the 69th day of storage) the content of the conjugated linoleic acids (CLA), as well as lowered the n6 to n3 fatty acids ratio. During the 69 days of storage, the content of carboxylic acids increased to more than 50%. In the period from the 42nd to 69th day of cheese storage, the content of butyric acid and hexanoic acids increased twofold, whereas that of octanoic acid increased more than tenfold. Fifty-four volatile compounds were identified in the cheese. The largest group was ketones (34%), whose level decreased during storage, with 2-butanone, 3-hydroxy- (acetoin) and 2-butanone predominating. The research found that due to their low odor threshold, carboxylic acids may have negatively affected the flavor profile of the cheese.

## 1. Introduction

The cheese market is one of the most dynamic food sectors. According to FAO data [1], global cheese production in 2000–2014 rose from 14.6 to more than 20 million tons, and cheese from cow milk from 13.2 to 18.7 million tons. About one third of cow milk produced globally is processed into a wide assortment of cheeses [2]. The vast majority of them (about 80%) are produced in industrial conditions from the milk of international breeds [3]. However, there is also a wide assortment of artisanal cheese produced in small processing facilities or directly on farms employing a semi-intensive or extensive milk production system. Many such farms keep cows of local breeds, whose diets are based on on-farm feedstuffs, primarily from permanent grassland [4]. Cheese production at the level of the farm or small manufacturing facilities can increase the income of these farms [5]. An increasing number of cheeses made from the milk of local breeds using a specific production technique are granted PDO or PGI certification. These indications confirm for the consumer that the product was produced using milk obtained via a farming system mainly based on natural pasture, and possesses characteristics resulting from tradition and unchanging local production [6,7]. The quality of dairy products is closely linked not only to the conditions in which they are produced, but also to the cow breed and feeding system (traditional vs. intensive) [8,9]. The production of traditional local food products is also associated with protection of animal health and welfare, environmental protection, biodiversity and sustainable development [10].

The quality of a finished product, including cheese, is determined by the raw material used. Research by many authors [8,9,11,12,13,14,15] has shown that the milk of animals raised in traditional and extensive systems, mainly based on pasture grazing, as well as the dairy products made from it, has a more beneficial fatty acid profile than milk produced in an intensive system. This is mainly due to a diet based on feedstuffs obtained from permanent grasslands, especially in the spring and summer, as well as to the elevation of these pastures above sea level [12,16,17,18,19].

There are four native cattle breeds in Poland: Polish Red, White-Backed, Polish Black-and-White and Polish Red-and-White. The oldest breed is Polish Red, whose population is largest in southern Małopolska (Lesser Poland). These cattle arrived in Poland together with population movements in the early 16th century. The breed is counted among small wild brachyceric (short-horned) cattle [14]. The milk of these cows is distinguished by very favorable parameters for cheese production [14,20,21], as well as high contents of compounds which are beneficial for human health (whey proteins, polyunsaturated fatty acids, and fat-soluble vitamins) [14,22,23].

During milk cheese making and the ageing of cheese, a number of physicochemical and microbiological transformations are involved. All biochemical reactions take place under the influence of indigenous milk enzymes, as well as clotting and bacterial enzymes [24]. The flavor of cheese is affected by the quality of the milk, the cheese making process and, most importantly, by the microbiological process [25,26,27]. The cheese environment is characterized by a complex population of bacteria causing numerous biochemical reactions resulting in the formation of aromatic volatile compounds [28,29]. The combination of volatile compounds and their interactions primarily contribute to the cheese aroma and flavor. Volatile substances influencing flavor include acids, ketones, alcohols, aldehydes, esters, and sulfur compounds. The flavor of cheese, in combination with its overall appearance and its odor and structure, has a decisive influence on consumer choices and preferences [30].

Smoking is one of the oldest methods of extending the shelf-life of food. Many chemical changes take place during this process (penetration of the chemical compounds contained in the smoke into the product, and the specific effects of smoke components on the product), as well as physical changes (mainly drying) [31]. Smoking reduces microbiological contamination and inhibits lipid oxidation (antioxidant effect of smoke). The components contributing to the specific flavor and odor bouquet, referred to as a smoky aroma, are volatile with water vapor, and are generally phenols, carbonyl compounds, and organic acids.

The aim of the study was to assess the proximate chemical composition and profile of fatty acids and volatile compounds during storage of smoked cheese made from the milk of cows of native Polish breeds included in a genetic resources conservation program.

## 2. Material and Methods

### 2.1. Cheese Production

The cheese was made in a local dairy in the village of Łużna (49°43′N 21°03′E), located in the Low Beskids in southern Poland. These are nonindustrialized areas with a low level of urbanization. The Dairy Cooperative in Łużna (near Gorlice) purchases milk from 150 local suppliers, with 75% of the milk delivered in tank trucks and 25% transported in a traditional manner, i.e., in milk churns. The dairy purchases 2.2 million liters of milk annually, most of which is from cows of the Polish Red breed. The remainder comes from the Polish Red-and-White and Polish Black-and-White breeds. All of these breeds are included in a genetic resources conservation program. The cows are kept in an extensive system, and their diet from spring to autumn is based on pasture grazing. The milk of these cows is used to produce smoked aged cheese.

The milk for cheese-making was pasteurized (75 °C, 15 s) and then cooled with ice water to 8 °C. Then, it was standardized at 2.9% fat level and heated to 30 °C to introduce the starter cultures (2%; Choozit Star 26 LYO 125 DCU, Danisco, København, Denmark; freeze-dried strains of *Streptococcus salivarius* subsp. *thermophilus*), together with rennet (1.4 mL/100 L of milk; MicroClage 2000 IMCU, GAP, Food Additives, Nowy Sącz, Poland). After about 40 min, the curd was cut into grains (approx. 3–6 mm diameter). The cut curd was mixed to obtain the appropriate whey acidity (4.8–5.0 °SH), and 25% of the whey was drained off. The resulting mass was dried for 5 min and then heated at 36–39 °C. The cheese was molded in molds able to hold 1.5 kg of cheese mass. The cheese mass packed into the molds was pressed with a gradual increase in pressure. The total pressing time was about 14 h. The cheese was brined in brine (NaCl 18–22%) at 12–14 °C for 7 h and dried in a ripening room for 3–4 days (12–14 °C; relative humidity approx. 85%). It was smoked in smoke obtained by burning beech wood chips at 35 °C. The smoking time did not exceed 5 h. When the cheese was ready for distribution (up to 4 days after manufacture, immediately after smoking), it was vacuum-packed in polyethylene bags. For the purposes of this study, three cheese making sessions were carried out in the dairy plant on three consecutive days. Every day, four cheeses were produced for a total of 12 cheeses. Four storage times were analyzed: one day after the drying and smoking phase, and then day 21, day 42, and day 69—the last day of the product’s shelf-life according to the manufacturer. The cheese was stored in refrigerated conditions (5 °C) until analysis.

### 2.2. Laboratory Analyses

#### 2.2.1. Acidity and Proximate Chemical Composition of Cheeses

The following parameters were determined in the cheese: active acidity (pH) (10 mL of distilled water preheated to 40 °C was added to 10 g of cheese and then the pH of a sample was measured using a pH meter Elmetron CP-401 (Zabrze, Poland), titratable acidity as lactic acid percentage (to convert acidity to lactic acid percentage, the result obtained in °SH was multiplied by the factor 0.0225), moisture content (by drying 3 g of sample at 102 °C until constant weight) and fat content by the Gerber method (PN-73/A-86232) [32]. The content of total nitrogen was determined by the Kjeldahl method, and the result was converted to the percentage content of protein using a conversion factor of 6.38 [33]. The analyses were carried out in triplicate on fresh, not frozen material on indicated storage days.

#### 2.2.2. Fatty Acid Profile

The fatty acid profile was determined following fat extraction using Schmid-Bondzynski-Ratzlaff method [34], with a slight modification involving replacing petroleum ether with n-hexane. The solvent was removed using a rotatory evaporator at 40 °C under reduced pressure. The extract was then dried at 40 °C under a stream of nitrogen, followed by frozen at −45 °C (LT U250, Nordic Lab, Vaerloese, Denmark). FA profile was performed within one week from fat extraction. Methyl esters of fatty acids (FAMEs) were prepared by the transmethylation of fat samples using a mixture of concentrated H_2_SO_4_ (95%) and methanol according to the AOCS Official Method Ce 2-66 [35]. FAMEs were analyzed by gas chromatography (GC) according to PN-EN ISO 12966-1:2015-01 [36] and PN-EN ISO 5508 [37] using the Varian GC 3900 (Walnut Creek, CA, USA) chromatograph equipped with a flame ionization detector (FID) and capillary column with stationary phase of high polarity (100 m × 0.25 mm I.D., film thickness 0.25 μm; CP 7420 Agilent Technologies, Santa Clara, CA, USA). The analysis was carried out in increasing temperature conditions. The temperature program was as follows: 50 °C for 1 min, 30 °C/min up to 120 °C, 2 °C/min up to 160 °C, 30 min holding, 1 °C/min up to 200 °C, 5 °C/min up 250 °C, 1 min holding time. The temperature of the injector and the detector was 260 °C and 270 °C, respectively; the carrier gas (hydrogen) flow rate was 2 mL/min, the size of the injected samples was 1 μL and the split ratio was 1:50. Fatty acids were identified by comparing the retention time of each FAME with those of fatty acid methyl ester standards (Supelco 37 Component FAME Mix CRM 47885 Supelco, CLA methyl ester O5632—Sigma-Aldrich, Branched Chain FAME Mixture BR2, BR3, BR4—Larodan AB, St. Louis, MO, USA) and literature data [38,39]. The results of the measurements were analyzed using Star GC Workstation v. 5.5. The analyses were carried out in triplicate.

The following groups of fatty acids and their ratios and indices were calculated:

- SCSFA—short- and medium-chain saturated fatty acids
- LCSFA—long-chain saturated fatty acids
- OCFA—odd-chain fatty acids
- BCFA—branched-chain saturated fatty acids
- SFA—saturated fatty acids
- MUFA—monounsaturated fatty acids
- PUFA—polyunsaturated fatty acids
- PUFA/SFA and MUFA/SFA ratios
- CLA—conjugated linoleic acid
- n-3 and n-6
- n-6/n-3 ratio
- DFA—desirable fatty acids—(MUFA + PUFA + C18:0)—according to Medeiros et al. [40]
- HSFA—hypercholesterolaemic saturated fatty acids—(C12:0 + C14:0 + C16:0)—according to Renna et al. [41]
- AI—atherogenic index = (C12:0 + 4 × C14:0 + C16:0) ÷ (MUFA + PUFA) according to Ulbricht and Southgate (1991) (qtd in:) Martínez Marín et al. [42],
- TI—thrombogenic index = (C14:0 + C16:0 + C18:0) ÷ (0.5 × MUFA + 0.5 × n6 + 3 × n3 + n3 ÷ n6) according to Ulbricht and Southgate (1991) (qtd in:) Hanuš et al. [43].

#### 2.2.3. Determination of Volatile Compounds

Cheese samples (200 g) on indicated storage days were collected and then frozen at −45 °C (LT U250, Nordic Lab, Vaerloese, Denmark). Analyses were performed within two weeks. Volatile compounds in the cheeses were determined by headspace solid phase micro-extraction (SPME) coupled with gas chromatography/mass spectrometry (GC/MS) (6890N GC, 5975 MS Agilent, Lexington, MA, USA) according to Antoniewska et al. [44]. Samples (5 g) were weighed in 20 mL vials, closed with silicone/PTFE seal caps, and heated at 40 °C for 30 min to stabilize the concentrations of volatiles in the headspace of the sample. Before analysis, a 50/30 μm SPME fiber coated with Divinylbenzene/Carboxen/Polydimethylsiloxane (DVB/CAR/PDMS) (Supelco, Bellefonte, PA, USA) was conditioned by heating in a gas chromatograph injector (GC) at a temperature of 270 °C for 30 min. Volatile compounds were extracted by exposing the SPME fiber in the headspace of the sample at 40 °C for 40 min. Then, the SPME fiber was quickly transferred to the GC injection port operating in split-less mode for 30 min.

Volatile compounds were separated on a HP-5MS column: 30 m × 0.25 mm × 0.25 μm film thickness, 5%-diphenyl-95%-polydimethylsiloxane (Agilent, Lexington, MA, USA). Helium was used as the carrier gas with a linear velocity 0.9 mL/min. Chromatographic separation was conducted under the following conditions: oven temperature held for 10 min at 38 °C, then increased to 200 °C (4 °C/min gradient) and held for 2 min, and finally, raised to 250 °C at 20 °C/min and held for 7 min. The mass detector was set as follows: interface temperature—150 °C; source temperature—230 °C; ionization energy—70 eV; and scan range—33–350 amu.

Data were acquired using MSD ChemStation software (Agilent, Lexington, MA, USA). Peaks were identified by comparing their mass spectra with the reference mass spectra of the NIST.08 and Wiley 8th Ed. libraries. Mass spectra of volatiles were confirmed by comparing linear retention indices (LRI) calculated (using AMDIS software, Lexington, MA, USA) relative to a series of standard alkanes (alkane standard solution C_8_–C_20_, Sigma-Aldrich, St. Louis, Missouri, USA) with the NIST.08 library LRI database. The areas of volatile compounds were calculated and expressed as percentages of the total peak area. Analyses were carried out in triplicate.

### 2.3. Statistical Analysis

One-way analysis of variance (ANOVA) of the results was performed using StatSoft Inc. STATISTICA, version 13 software [45]. Significance of differences between means for each parameter was determined by the Tukey test at a significance level of *p* < 0.05.

## 3. Results and Discussion

The data in Table 1 show that the active acidity (pH) of the cheese during ten-week storage remained at a similar level (5.35–5.38). A significant (*p* ≤ 0.01) increase was noted in the content of lactic acid at the end of the storage period (by over 20%). The content of dry matter, fat and protein did not change significantly during the 69-day storage period. The use of vacuum packing and packing materials with a high gas barrier slowed the transformations induced by the presence of oxygen, thereby preserving a relatively constant cheese composition throughout the storage period. Sıçramaz et al. [46] showed no significant changes in the content of dry matter, fat or total nitrogen content during 90-day storage of traditional Turkish Circassian cheese that was smoked and vacuum-packed. The titratable acidity of cheese stored at 5 °C increased by 29% (from 1.10 to 1.42%) during 90-day storage, a similar result to that obtained in the present study.

### 3.1. Fatty Acid Profile

No significant changes in the proportion of short- and medium-chain saturated fatty acids (Ʃ SCSFA) were noted in the cheese fat during storage (Table 2). The proportion of long-chain fatty acids (Ʃ LCSFA) increased by just 0.6% (after 42 days of storage). After 21 days, there was a significant (*p* < 0.05) and steady decrease (up to day 69) in the proportion of odd-chain (by about 36%) and branched-chain (by about 17%). At the same time, the proportion of unsaturated acids increased. Among unsaturated fatty acids (*p* < 0.05), however, there was a significant increase in the proportion of monounsaturated fatty acids (by 5%) and a decrease in polyunsaturated fatty acids of nearly 12%. Storage was also found to exert a negative effect on the CLA content in the cheese, as it declined steadily from the first week (by 47%). These changes may be due to lipid oxidation, to which unsaturated fatty acids are particularly susceptible, which altered the proportions of individual groups of fatty acids. Laskaridis et al. [47] showed an increase in the content of unsaturated fatty acids in the fat of Feta cheese made from sheep milk during the initial period of storage, with a subsequent substantial decline in the content of MUFA and PUFA after about 40 days, as in the present study. In the case of both types of cheese, on the last day of the study (day 69 for cheese from the milk of Polish native cow breeds; day 65 for Feta), an increase in MUFA and a decrease in PUFA were observed relative to day 1. Fletouris et al. [48] investigated changes in the fatty acid composition of Graviera Agraphon cheese produced by a local processing facility following a three-month ageing period and storage for 60 days in modified-atmosphere packaging. After 30 days, they observed a significant (*p* < 0.05) increase in the level of SFA, after which it remained stable, while the total concentration of MUFA and PUFA decreased up to 30 days of storage, and then remained stable. Conjugated linoleic acid (CLA) is naturally present in cheese, its main source being the milk it was made from [49]. According to Lobos-Ortega et al. [50], the CLA content in cheese is highly varied and depends primarily on how the animals are fed. The highest CLA content is found when animals have direct access to the pasture. Natural mountain pastures with greater floristic richness, and above all, the presence of more species of meadow plants and herbs, are particularly beneficial for the CLA content in milk and subsequently in cheese [12]. The bacterial strains used in cheese production also determine its concentration, because they are responsible for converting linoleic acid into conjugated forms (CLA) [50]. CLA content in dairy products is also influenced by pH and the ripening period [50]. However, opinions differ as to the effect of cheese storage on its content of CLA. Laskaridis et al. [47] reported that the content of CLA in Feta cheese fell during 42 days of storage, while according to Fletouris et al. [48], it decreased in Graviera Agraphon after just 30 days. A decrease in CLA content during storage was also noted by Fletouris et al. [48] in cheese produced from sheep milk and packed in a modified atmosphere, as well as by Gulzar et al. [51] in Mozzarella made from buffalo milk. Shantha et al. [52], on the other hand, showed no increase or decrease in CLA in Mozzarella or Cheddar stored for 32 weeks or in Gouda stored for 30 weeks. Research confirms that the storage of cheese in plastic foil bags in refrigerated conditions causes oxidation of acids, particularly CLA, thus reducing its content. Oxidation of acids is promoted not only by the transparent foil, but also the amount of free space filled with air in the packaging [53].

In the present study (Table 2), there was a small increase in the content of desirable fatty acids (DFA) after 21 days of storage, by nearly 2% on the final day. Thus, there was an accompanying decrease in hypercholesterolaemic saturated fatty acids (HSFA) during storage, which reached their lowest level on the last day (1% decrease). Similar changes in the content of hypercholesterolaemic saturated fatty acids were found by Laskaridis et al. in Feta cheese [47]. The reverse tendency was observed by Fletouris et al. [48] in their analysis of changes in the fatty acid profile of Graviera Agraphon cheese during storage, as the content of DFA fell, and that of hypercholesterolaemic saturated fatty acids (HSFA) rose. The content of n-3 and n-6 fatty acids decreased steadily during storage, particularly n-3 (by more than 60%), while the n-6 content fell by one third. The most favorable n-6/n-3 ratio (0.22) persisted up to the 21st day of storage, after which irregular changes in these groups of acids resulted in the least favorable ratio (0.76) on day 42 (Table 2). The ratio was similar to that reported by Romanzin et al. [54] in Montasio cheese made from the milk of Simmental cows and evaluated at 60 days of storage (0.74).

To address the difficulty of determining the nutritional value of fatty acids, Ulbricht and Southgate proposed the atherogenic index (AI) and thrombogenic index (TI) for hypercholesterolaemic fatty acids (C12:0; C14:0 and C16:0), which have negative effects on human health. The higher the values of these indices in milk fat, the higher the risk of cardiovascular disease [40]. In the present study, the atherogenic and thrombogenic indices of the cheese during storage were relatively low. The atherogenic index underwent small but statistically significant changes (*p* < 0.05), amounting to slightly over 2 for the entire storage period, while the thrombogenic index increased by about half during the second half of the storage period (Table 2). The values obtained for the atherogenic and thrombogenic indices are much lower in the case of cheese made from the milk of cows kept in an intensive system. The atherogenic index for Italian artisanal Canestrato cheese made from the milk of Friesian cows was 4.16, and the thrombogenic index was 5.02 [55]. In the case of Cacioricotta cheese from the milk of Simmental cows, the values of these indices were 2.91 (AI) and 3.31 (TI) [56].

### 3.2. Profile of Volatile Compounds

Volatile compounds (VCs) arise in many biochemical processes, including lipolysis, proteolysis and the metabolism of fatty acids or amino acids [57]. Smoking is one of the oldest methods of preserving food, and additionally affects the palatability of food products. The smoking process, depending on how it is carried out, initiates a number of transformations of the components of cheese, thereby modifying its sensory properties. The available literature lacks studies on changes in the profile of volatile compounds during the storage of smoked cheese.

Table 3 shows the results relating to the profile of volatile compounds determined in the cheeses under examination. There were 53 volatile compounds identified in the cheese, including ketones (18), aldehydes (7), alcohols (6), carboxylic acids (4), furan compounds (4), hydrocarbons (3), lactones (3), esters (3) and other compounds (5) (Table 3). The VC profile of the cheese underwent quantitative and qualitative changes during the ten weeks of storage.

Ketones were the largest group (34% of all compounds). They are formed as a result of ß-oxidation and decarboxylation of free fatty acids, and are among the most important compounds determining the palatability of cheese [58]. Their content in the cheese decreased significantly (*p* < 0.05) during storage (from 32.68% in the fresh cheese to 14.29% at the end of the product’s shelf-life). The dominant compounds in this group were 2-butanone, and 2-butanone, 3-hydroxy- (acetoin). Their content in the fresh cheese was 10.38% and 9.75%, respectively, dropping sharply in successive weeks of storage to 2.61% and 3.53%. Acetoin is an odorless compound that is easily oxidized to diacetyl, i.e., 2,3-butanedione, which has a pleasant, creamy, buttery aroma, or reduced to 2,3-butanediol, depending on the reducing/oxidizing conditions of the environment. These compounds are synthesized by bacteria of the genera *Lactococcus* and *Leuconostoc* from pyruvic acid. Acetoin is formed directly from pyruvate in a reaction catalyzed by acetolactate decarboxylase, with α-acetolactate formed as a precursor [59]. Acetoin is a volatile compound characteristic of dairy products, and is present in high concentrations only in the first few days of ripening [10]. According to Carpino et al. [60], 2-butanone, 3-hydroxy- is one of the compounds that distinguishes cheese produced from the milk of pasture-grazed cows from cheese made from the milk of cows fed preserved feed (maize meal or maize silage).

The level of aldehydes in the cheese changed as well. These compounds are present in cheese temporarily, as they are quickly reduced to primary alcohols or oxidized to acids or hydroperoxides, which are then converted to hydrocarbons, alcohols and carbonyl compounds. They have low odor thresholds, so they can significantly affect the smell of cheese [61,62]. In the present study, seven aldehydic compounds were identified in the cheese, two of which appeared in the sixth week of storage. The total content of aldehydes decreased steadily from the moment of production (4.85%) to the sixth week of storage (3.40%), and then increased (to 5.11%). The dominant aldehydes in the fresh cheese included 3-methylbutanal and benzaldehyde—compounds produced by the conversion of the amino acids leucine and phenylalanine, respectively. They are responsible for fruity (sweet cherry), nutty (bitter almond), malty and cheesy off-flavors.

Some aldehydes are indicators of lipid peroxidation in dairy products. Hexanal (present in the cheese from day 42 of storage) is formed as a result of oxidation of oleic and linoleic acids, and pentanal (present in the cheese from the moment of production) through oxidation of arachidic and linoleic acid. These compounds are responsible for metallic and cardboard off-flavors. The appearance of these compounds deriving from the oxidation process of polyunsaturated fatty acids could be related to the simultaneous decrease of the latter highlighted in cheeses after the sixth week (42 days) of storage. Unsaturated fatty acids, as highlighted above, are present in higher concentrations in the milk and derived products of pasture-grazed cows than in the milk of cows kept indoors [25].

Among alcohols, six compounds were identified (Table 3). The total content of alcohols increased up to day 42 of storage, and then decreased substantially (by 36%). The dominant compound in this group was ethanol. It is formed as a result of the fermentation of lactose or the catabolism of alanine, and plays an important role in ester formation [61].

The content of carboxylic acids in the cheese remained stable until day 42 of storage (18.46–21.35%), after which a marked increase in their concentration was observed (to 30.71%). The dominant compound throughout the storage period was acetic acid (responsible for sharp, vinegar notes). At the end of the shelf-life of the cheese, the content of butyric acid (imparting sharp, rancid and sour notes) and hexanoic acid (fatty, sharp, and goat notes) rose more than twofold, and that of octanoic acid (sweet, goat notes) more than tenfold compared to day 42 of storage. Due to the low odor thresholds of short- and medium-chain carboxylic acids, they influence the flavor profile of cheese. Carboxylic acids are also precursors of other aromatic compounds, including alcohols, lactones and esters [63]. However, the results of sensory analysis did not show any significant effect of storage time on cheese flavour, aroma, colour and texture (Figure S1).

Four furan compounds were identified, but not all were present in the cheese during the entire storage period; 2-furanmethanol was present in the greatest quantities, and was the only compound that remained in the cheese throughout its shelf-life. This compound may have appeared as a result of thermal degradation of the polysaccharides present in the wood used to smoke the cheese or during the pasteurization of milk used for the production of cheeses. Furans usually result from the thermal degradation of fructose in the presence of amines and amino acids in the Maillard reaction. They are responsible for pleasant roasted and caramel notes [64].

Three hydrocarbons were identified, but only one (toluene) was present in the cheese in small amounts up to the tenth week of storage. Cyclohexene, 1-methyl-4-(1-methylethenyl) was present only in fresh cheese (0.16%), while cyclopentane appeared only on the final day of the study (0.84%). Toluene may have been formed by the degradation of carotene present in the milk of pasture-grazed cows.

Three lactones were identified as well, i.e., γ-butyrolactone, α-methyl-γ-crotonolactone and maple lactone, with only γ-butyrolactone present in the fresh cheese (0.29%). Palencia et al. [65] reported that γ-lactones are characteristic only of smoked cheese. According to Bovolenta et al. [63], γ-butyrolactone is present in higher concentrations in cheese made from the milk of cows grazing on pastures with little botanical diversity compared to species-rich pastures.

The presence of esters in cheese may be due to reactions of free fatty acids with alcohols [10]. Among the three compounds identified, two (butanoic acid, ethyl ester and 2(3H)-furanone, 5-methyl) were present in the cheese throughout the storage period. At the end of the shelf-life of the cheese, hexanoic acid and ethyl ester were detected. Esters are important compounds influencing the aroma bouquet of cheese due to their low odor thresholds. The effect of the esters on the aromatic bouquet of cheeses may be positive (they are able to mask unpleasant off-flavors and off-odors resulting from high concentrations of short-chain fatty acids, methyl ketones, and amines) or negative (high concentrations of esters can cause defects in the palatability of cheese, i.e., fruity off-flavors) [58].

Among substances that do not belong to any of the groups mentioned above, four phenolic compounds and one terpene were identified. Phenol; phenol, 3-methyl-; phenol, 2-methoxy; and 1-hydroxy-2-methoxy-4-methylbenzene are substances originating in smoke that settle on the surface of cheese during smoking and then migrate into them, imparting a smoky aroma. Their content in the cheese increased up to the sixth week of storage. α-Pinene was present in the cheese at similar levels from the third week of storage (0.12–0.16%). Terpenes are an important component of alpine cheese. They result from the secondary metabolism of pasture plants, from which they pass into milk and ultimately into cheese produced from it [63].

## 4. Conclusions

To sum up, the rennet cheese made from the milk of native cow breeds grazing in the natural meadows of the Low Beskids, smoked, vacuum-packed and stored in refrigerated conditions for 69 days (their shelf-life according to the manufacturer), retained a relatively constant chemical composition throughout the storage period. Moreover, up to 21 days of storage, no significant changes took place in the fatty acid profile or volatile compound profile. After that time, the profile of fatty acids became less favorable in terms of health-promoting properties (i.e., a decrease in the share of odd-chain, branched-chain, PUFA including CLA and an increase in the n6/n3 acid and thrombogenic index ratios). Among volatile compounds, ketones were the largest group (34%). Their content decreased significantly (*p* < 0.05) during storage (changes mainly in the levels of 2,3-butanedione, 2-butanone and 2-butanone, 3-hydroxy-). During the 69 days of storage, the content of carboxylic acids increased by more than 50%. At the end of the shelf-life of the cheese, the content of butyric acid (imparting sharp, rancid and sour notes) and hexanoic acid (fatty, sharp, and goat notes) rose more than twofold, and that of octanoic acid (sweet, goat notes) more than tenfold relative to day 42 of storage. Due to the low detection thresholds of carboxylic acids, they may have adversely affected the flavor profile of the cheese.

Regional cheese from Łużna is gaining a growing group of consumers, including from Krakow and its vicinity, for whom the repeatable quality of the product and its high nutritional and sensory value are extremely important. Therefore, it would be advisable for manufacturers to take certain measures to ensure that the high quality of the product persists throughout its shelf-life. It would be worth considering packing this cheese in a modified atmosphere in order to limit biochemical transformations leading to negative changes in the profiles of fatty acids and volatile compounds. Otherwise, the stated shelf-life should be reduced.

## Figures and Tables

**Table 1:** Evolution of physicochemical properties of smoked cheese during 69 days of storage (mean ± SD, *n* = 3).

Parameters	Smoked Cheese			
Day	1 (day 1)	2 (day 21)	3 (day 42)	4 (day 69)
pH	5.36 ± 0.49	5.36 ± 0.52	5.35 ± 0.47	5.38 ± 0.33
Lactic cid [%]	1.26 ^a^ ± 0.09	1.24 ^a^ ± 0.06	1.24 ^a^ ± 0.05	1.51 ^b^ ± 0.09
Dry matter [%]	61.55 ± 0.73	61.63 ± 0.57	61.81 ± 0.61	62.34 ± 0.74
Fat [%]	28.25 ± 0.61	28.75 ± 0.25	28.75 ± 0.54	29.0 ± 0.29
Protein [%]	27.08 ± 0.25	26.69 ± 0.31	27.98 ± 0.28	26.90 ± 0.24

a, b—differences significant at *p* < 0.05.

**Table 2:** Evolution of fatty acid profile [g/100 g fatty acids] during storage of cheese for 69 days (mean ± SD, *n* = 3).

Parameters	Smoked Cheese			
Day	1 (day 1)	2 (day 21)	3 (day 42)	4 (day 69)
Ʃ SCSFA	10.02 ± 0.03	10.13 ± 0.03	10.14 ± 0.12	10.16 ± 0.11
Ʃ LCSFA	50.73 ± 0.28	50.70 ± 0.38	51.02 ± 0.41	50.93 ± 0.39
Ʃ OCFA	2.18 ^c^ ± 0.05	2.21 ^d^ ± 0.07	1.46 ^b^ ± 0.03	1.41 ^a^ ± 0.07
Ʃ BCFA	2.22 ^b^ ± 0.01	2.23 ^b^ ± 0.06	1.89 ^a^ ± 0.07	1.84 ^a^ ± 0.06
Ʃ SFA	65.41 ^c^ ± 0.47	65.53 ^d^ ± 0.59	64.81 ^b^ ± 0.44	64.63 ^a^ ± 0.46
Ʃ UFA	34.59 ^b^ ± 0.61	34.46 ^a^ ± 0.54	35.19 ^c^ ± 0.38	35.37 ^d^ ± 0.42
Ʃ MUFA	29.59 ^b^ ± 0.53	29.28 ^a^ ± 0.18	30.92 ^d^ ± 0.29	30.80 ^c^ ± 0.33
Ʃ PUFA	5.01 ^c^ ± 0.03	5.19 ^d^ ± 0.11	4.27 ^a^ ± 0.07	4.57 ^b^ ± 0.10
Ʃ CLA	2.64 ^c^ ± 0.08	2.36 ^b^ ± 0.06	1.49 ^a^ ± 0.02	1.39 ^a^ ± 0.04
DFA	45.31 ^b^ ± 0.47	45.09 ^a^ ± 0.62	45.76 ^c^ ± 0.35	45.92 ^d^ ± 0.54
n-3	1.37 ^d^ ± 0.14	1.20 ^c^ ± 0.08	0.36 ^a^ ± 0.01	0.52 ^b^ ± 0.08
n-6	0.30 ^b^ ± 0.08	0.26 ^b^ ± 0.02	0.28 ^b^ ± 0.02	0.20 ^a^ ± 0.03
n-6/n-3	0.22 ^a^ ± 0.07	0.22 ^a^ ± 0.03	0.76 ^c^ ± 0.04	0.39 ^b^ ± 0.06
HSFA	42.33 ^c^ ± 0.22	42.38 ^d^ ± 0.38	42.07 ^b^ ± 0.27	41.97 ^a^ ± 0.31
AI	2.08 ^b^ ± 0.09	2.11 ^c^ ± 0.07	2.04 ^a^ ± 0.07	2.03 ^a^ ± 0.05
TI	1.80 ^a^ ± 0.07	1.84 ^b^ ± 0.04	2.33 ^d^ ± 0.07	2.14 ^c^ ± 0.08

a, b, c, d—the averages given with different letters in the same row indicate significant differences at the 0.05 level from each other.

**Table 3:** Area percentage of the volatile compounds detected in cheeses during 69-day storage (mean ± SD, *n* = 3).

**Day**	**RI**	**Smoked Cheese**			
		**1 (day 1)**	**2 (day 21)**	**3 (day 42)**	**4 (day 69)**
Alcohols					
Ethanol	550	5.31 ^a^ ± 0.33	6.36 ^a^ ± 0.96	10.51 ^b^ ± 1.70	5.79 ^a^ ± 1.64
Cyclobutanol	665	0.28 ^a^ ± 0.3	1.01 ^b^ ± 0.01	Nd	0.38 ^a^ ± 0.02
2,3-Butanediol	777	Nd	Nd	Nd	0.68 ± 0.39
Butanol, 3-methyl-	725	3.26 ^b^ ± 0.37	3.75 ^b^ ± 1.15	3.02 ^ab^ ± 0.16	1.83 ^a^ ± 0.64
1,3-Butanediol	793	Nd	0.53 ± 0.11	Nd	Nd
1-Hexanol	865	0.19 ^a^ ± 0.03	1.28 ^b^ ± 0.17	Nd	Nd
Total		9.04 ^a^ ± 0.27	12.93 ^b^ ± 0.48	13.53 ^b^ ± 0.92	8.68 ^a^ ± 0.69
Ketones					
2-Propanone	558	3.62 ± 0.26	3.52 ± 0.14	3.62 ± 0.73	2.45 ± 0.88
2,3-Butanedione	593	1.62 ^b^ ± 0.14	1.06 ^a^ ± 0.07	1.09 ^a^ ± 0.13	0.86 ^a^ ± 0.11
2-Butanone	598	9.75 ^b^ ± 0.10	3.76 ^a^ ± 0.16	2.44 ^a^ ± 0.42	3.53 ^a^ ± 0.58
1-Hydroxyacetone	663	2.00 ^c^ ± 0.19	1.61 ^b^ ± 0.13	1.70 ^b^ ± 0.14	1.17 ^a^ ± 0.10
2-Pentanone	682	1.00 ± 0.67	1.05 ± 0.47	Nd	Nd
2-Butanone, 3-hydroxy-	706	10.38 ^c^ ± 1.51	4.27 ^b^ ± 0.20	4.81 ^b^ ± 0.08	2.61 ^a^ ± 0.53
Cyclopentanone	783	0.45 ± 0.34	0.72 ± 0.17	0.72 ± 0.27	Nd
2-Cyclopenten-1-one	827	Nd	1.27 ± 0.19	1.05 ± 0.17	Nd
2-Propanone, 1-(acetoloxy)-	873	0.84 ^ab^ ± 0.09	1.23 ^b^ ± 0.06	0.64 ^a^ ± 0.21	Nd
2H-Pyran-3(4H)-one, dihydro-	892	Nd	0.21 ± 0.07	0.16 ± 0.03	Nd
2-Heptanone	890	0.56 ^b^ ± 0.05	0.66 ^b^ ± 0.01	0.31 ^a^ ± 0.06	0.62^b^ ± 0.04
2-Methyl-2-cyclopentenone	904	0.56 ^a^ ± 0.03	0.73 ^ab^ ± 0.08	0.55 ^a^ ± 0.08	0.83 ^b^ ± 0.05
3-Methyl-2-cyclopenten-1-one	964	0.76 ± 0.14	0.85 ± 0.08	0.85 ± 0.04	0.75 ± 0.07
2-Butanone, 1-(acetyloxy)-	973	Nd	0.64 ± 0.14	Nd	Nd
2-Methyltetrahydrothiophen-3-one	985	0.17 ± 0.07	Nd	Nd	0.16 ± 0.03
2,3-Dimethyl-2-cyclopenten-1-one	1038	0.45 ^a^ ± 0.14	0.86 ^c^ ± 0.19	0.66 ^b^ ± 0.15	0.58 ^a^ ± 0.13
3-Ethyl-2-cyclopenten-1-one	1075	Nd	0.22 ± 0.06	0.25 ± 0.08	0.14 ± 0.06
2-Nonanone	1093	0.52 ± 0.11	Nd	Nd	0.59 ± 0.16
Total		32.68 ^d^ ± 0.35	22.66 ^c^ ± 0.24	18.85 ^b^ ± 0.35	14.29 ^a^ ± 0.27
Aldehydes					
Pentanal	695	1.43 ^b^ ± 0.64	2.01 ^c^ ± 0.75	0.72 ^a^ ± 0.13	2.05 ^c^ ± 0.11
3-Methylbutanal	643	2.05 ^c^ ± 0.02	1.22 ^b^ ± 0.49	0.79 ^a^ ± 0.51	1.05 ^a^ ± 0.06
Hexanal	798	Nd	Nd	0.88 ^a^ ± 0.08	1.31 ^b^ ± 0.09
Heptanal	902	0.18 ± 0.09	0.16 ± 0.09	0.09 ± 0.03	0.12 ± 0.03
Benzaldehyde	959	0.93 ± 0.66	0.49 ± 0.19	0.40 ± 0.10	0.37 ± 0.13
Nonanal	1104	0.26 ^a^ ± 0.02	0.34 ^b^ ± 0.02	0.37 ^b^ ± 0.02	0.21 ^a^ ± 0.02
Decanal	1206	Nd	Nd	0.15 ± 0.06	Nd
Total		4.85 ^b^ ± 0.31	4.22 ^b^ ± 0.29	3.40 ^a^ ± 0.14	5.11 ^c^ ± 0.08
Carboxylic acids					
Acetic acid	622	11.44 ^ab^ ± 0.39	10.03 ^a^ ± 0.68	10.00 ^a^ ± 0.21	12.10 ^b^ ± 0.56
Butanoic acid	816	4.93 ^a^ ± 0.59	8.24 ^b^ ± 0.76	5.95 ^ab^ ± 1.01	12.02 ^c^ ± 0.34
Hexanoic acid	1005	3.62 ^b^ ± 0.05	2.98 ^a^ ± 0.38	2.48 ^a^ ± 0.29	6.17 ^c^ ± 0.66
Octanoic acid	1182	0.10 ± 0.04	0.10 ± 0.03	0.03 ± 0.01	0.42 ± 0.09
Total		20.09 ^ab^ ± 0.28	21.35 ^b^ ± 1.83	18.46 ^a^ ± 4.42	30.71 ^c^ ± 0.43
Furan compounds					
2-Furaldehyde or furfural	828	2.0 ^c^ ± 0.02	0.26 ^a^ ± 0.05	0.67 ^b^ ± 0.11	Nd
2-Furanmethanol	856	1.90 ^a^ ± 0.10	2.99 ^b^ ± 0.21	3.13 ^ab^ ± 0.31	3.49 ^c^ ± 0.57
Acetylfuran	911	Nd	0.25 ± 0.08	0.16 ± 0.09	0.18 ± 0.06
2-Furancarboxaldehyde,5-methyl-	965	Nd	Nd	0.14 ± 0.02	Nd
Total		3.90 ^b^ ± 0.07	3.50 ^a^ ± 0.10	4.10 ^c^ ± 0.09	3.67 ^a^ ± 0.33
Hydrocarbons					
Cyclopentane	581	Nd	Nd	Nd	0.84 ± 0.21
Toluen	754	0.76 ± 0.29	0.74 ± 0.26	0.59 ± 0.26	1.00 ± 0.24
Cyclohexene, 1-methyl-4-(1-methylethenyl)	811	0.16 ± 0.02	Nd	Nd	Nd
Total		0.92 ^b^ ± 0.16	0.74 ^a^ ± 0.26	0.59 ^a^ ± 0.06	1.84 ^c^ ± 0.19
Lactones					
γ-butyrolactone	913	0.29 ^a^ ± 0.05	0.38 ^b^ ± 0.05	0.55 ^c^ ± 0.16	0.47 ^c^ ± 0.11
α-Methyl-γ-crotonolactone	980	Nd	0.47 ± 0.01	0.47 ± 0.02	0.46 ± 0.06
2-Hydroxy-1-methylcyclopenten-3-one or Maple lactone	1027	Nd	0.64 ^a^ ± 0.12	1.04 ^b^ ± 0.19	0.65 ^a^ ± 0.21
Total		0.29 ^a^ ± 0.05	1.49 ^b^ ± 0.06	2.06 ^c^ ± 0.11	1.58 ^b^ ± 0.14
Esters					
Butanoic acid, ethyl ester	802	1.22 ± 0.11	1.86 ± 0.16	1.30 ± 1.03	1.91 ± 0.47
Hexanoic acid, ethyl ester	1001	Nd	Nd	Nd	2.28 ± 0.64
2(3H)-Furanone, 5-Methyl-	943	0.06 ^a^ ± 0.01	0.38 ^c^ ± 0.21	0.13 ^b^ ± 0.02	0.16 ^b^ ± 0.02
Total		1.28 ^a^ ± 0.09	2.24 ^b^ ± 0.12	1.43 ^a^ ± 0.54	4.35 ^c^ ± 0.33
Other compounds					
α-Pinene	931	Nd	0.16 ± 0.09	0.12 ± 0.02	0.16 ± 0.03
Phenol	988	0.36 ^a^ ± 0.01	0.55 ^b^ ± 0.02	0.82 ^c^ ± 0.01	0.32 ^a^ ± 0.02
Phenol, 3-methyl-	1059	0.19 ^a^ ± 0.08	0.89 ^b^ ± 0.46	1.82 ^c^ ± 0.33	0.15 ^a^ ± 0.02
Phenol, 2-methoxy-	1088	0.83 ^a^ ± 0.03	2.86 ^b^ ± 0.07	4.31 ^c^ ± 0.35	1.01 ^a^ ± 0.17
1-Hydroxy-2-methoxy-4-methylbenzene	1192	0.22 ^a^ ± 0.05	Nd	1.56 ^b^ ± 0.26	0.21 ^a^ ± 0.08
Total		3.46 ^b^ ± 0.10	6.23 ^c^ ± 0.17	8.63 ^d^ ± 0.21	1.85 ^a^ ± 0.07

a, b, c, d—the averages given with different letters in the same row indicate significant differences at the 0.05 level from each other; Nd—not detected; RI—Retention index.

## Supplementary Materials

The following are available online at https://www.mdpi.com/2076-2615/10/11/2103/s1, Figure S1: Results of sensory evaluation of cheeses during storage.
