# Supplementary material for: Changes in Fatty Acid and Volatile Compound Profiles during Storage of Smoked Cheese Made from the Milk of Native Polish Cow Breeds Raised in the Low Beskids
Source: Animals (Basel). 2020 Nov 12;10(11):2103. doi: 10.3390/ani10112103 (PMC7696590; doi:10.3390/ani10112103)
Supplement: Supplementary file 1 [file animals-10-02103-s001.pdf]

animals

MDPI

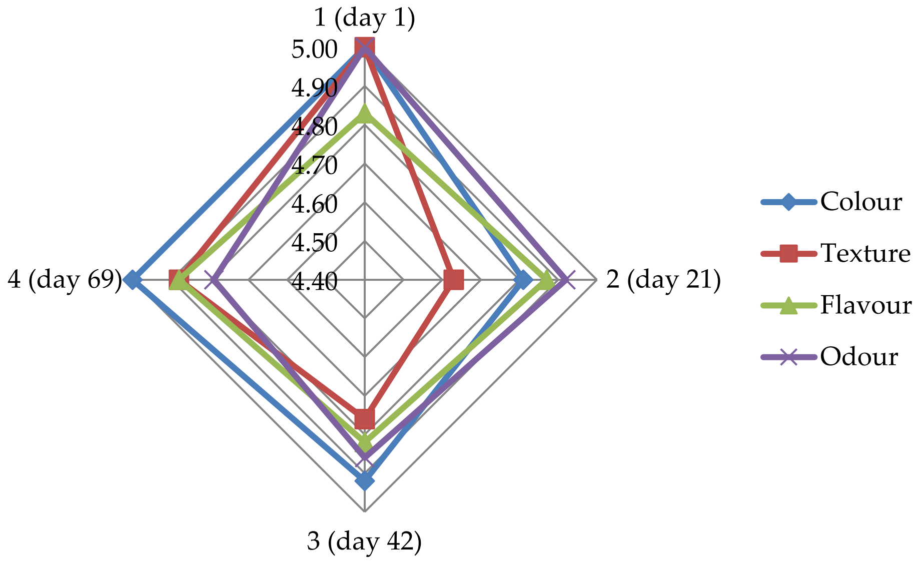

Figure S1. Results of sensory evaluation of cheeses during storage: 1—one day after the drying and smoking phase, 2—day 21, 3—day 42, 4—day 69. The scale of flavour and odour intensity had 5 basic levels: 5 – very strong, 4 – strong, 3—moderate, 2—weak, 1—slightly perceptible, or threshold. The levels adopted for cheese colour were 5—intense and uniform, 4 medium intensity and uniform, 3 – intense and moderately uniform, 2—low intensity and low uniformity, 1—not intense and not uniform. The levels for texture were 5—very hard, 4—hard, 3—somewhat hard, 2—neither hard nor soft, 1—somewhat soft. A sensory evaluation was carried out by a panel of eight people who had previously passed a general sensory skills test. Before evaluation, the cheese samples were cut into 1.5 cm cubes, coded, and left at room temperature for 1 h. Then they were presented to the testers together with a sensory evaluation questionnaire.

Publisher's Note: MDPI stays neutral with regard to jurisdictional claims in published maps and institutional affiliations.

© 2020 by the authors. Licensee MDPI, Basel, Switzerland. This article is an open access article distributed under the terms and conditions of the Creative Commons Attribution (CC BY) license (http://creativecommons.org/licenses/by/4.0/).
